# Development of Digital Strategies for Reducing Sedentary Behavior in a Hybrid Office Environment: Modified Delphi Study

**DOI:** 10.2196/59405

**Published:** 2025-04-08

**Authors:** Iris Parés-Salomón, Cristina Vaqué-Crusellas, Alan Coffey, Bette Loef, Karin I Proper, Anna M Señé-Mir, Anna Puig-Ribera, Kieran P Dowd, Judit Bort-Roig

**Affiliations:** 1 Faculty of Health Sciences and Welfare University of Vic – Central University of Catalonia (UVic-UCC) Vic Spain; 2 Sport and Physical Activity Research Group Institute for Research and Innovation in Life and Health Sciences in Central Catalonia (IRIS-CC) University of Vic – Central University of Catalonia (UVic-UCC) Vic Spain; 3 Research group on Methodology, Methods, Models and Outcomes of Health and Social Sciences (M3O) Institute for Research and Innovation in Life Sciences and Health in Central Catalonia (IRIS-CC) Vic Spain; 4 SHE Research Centre Department of Sport and Health Sciences Technological University of the Shannon Athlone, County Westmeath Ireland; 5 Centre for Prevention, Lifestyle and Health National Institute for Public Health and the Environment Bilthoven The Netherlands; 6 Department of Public and Occupational Health Amsterdam UMC, Vrije Universiteit Amsterdam Amsterdam Public Health Research Institute Amsterdam The Netherlands; 7 Sport and Physical Activity Research Group Sport and Physical Activity Studies Centre University of Vic-Central University of Catalonia Vic Spain

**Keywords:** sedentary behavior, office work, home office, hybrid work, technology, Delphi

## Abstract

**Background:**

Hybrid work is the new modus operandi for many office workers, leading to more sedentary behavior than office-only working. Given the potential of digital interventions to reduce sedentary behavior and the current lack of studies evaluating these interventions for home office settings, it is crucial to develop digital interventions for such contexts involving all stakeholders.

**Objective:**

This study aimed to reach expert consensus on the most feasible work strategies and the most usable digital elements as a delivery method to reduce sedentary behavior in the home office context.

**Methods:**

A modified Delphi study including 3 survey rounds and focus groups was conducted to achieve consensus. The first Delphi round consisted of two 9-point Likert scales for assessing the feasibility of work strategies and the potential usefulness of digital elements to deliver the strategies. The work strategies were identified and selected from a scoping review, a systematic review, and 2 qualitative studies involving managers and employees. The median and mean absolute deviation from the median for each item are reported. The second round involved 2 ranking lists with the highly feasible strategies and highly useful digital elements based on round 1 responses to order the list according to experts’ preferences. The weighted average ranking for each item was calculated to determine the most highly ranked work strategies and digital elements. The third round encompassed work strategies with a weight above the median from round 2 to be matched with the most useful digital elements to implement each strategy. In total, 4 focus groups were additionally conducted to gain a greater understanding of the findings from the Delphi phase. Focus groups were analyzed using the principles of reflexive thematic analysis.

**Results:**

A total of 27 international experts in the field of occupational health participated in the first round, with response rates of 86% (25/29) and 66% (19/29) in rounds 2 and 3, respectively, and 52% (15/29) in the focus groups. Consensus was achieved on 18 work strategies and 16 digital elements. Feedback on activity progress and goal achievement; creating an action plan; and standing while reading, answering phone calls, or conducting videoconferences were the most feasible work strategies, whereas wrist-based activity trackers, a combination of media, and app interfaces in smartphones were the most useful digital elements. Moreover, experts highlighted the requirement of combining multiple levels of strategies, such as social support, physical environment, and individual strategies, to enhance their implementation and effectiveness in reducing sedentary behavior when working from home.

**Conclusions:**

This expert consensus provided a foundation for developing digital interventions for sedentary behavior in home office workers. Ongoing interventions should enable the evaluation of feasible strategies delivered via useful digital elements in home office or hybrid contexts.

## Introduction

### Background

Workplace sedentary behavior (SB) is associated with poorer health and work-related outcomes, such as musculoskeletal disorders, reduced mental and occupational well-being, job dissatisfaction, and fatigue [1-4]. According to the World Health Organization, the workplace is one of the main settings to reduce SB in the adult population [5], with office workers, who spend an average of 77% of their working day sitting [6], being a key target group [7]. After the COVID-19 pandemic, hybrid work, alternating work in the office and at home, has become the new work paradigm for many desk-based jobs [8-10]. Hybrid work results in even higher levels of SB than working solely in the office [11], increasing the risk of musculoskeletal health problems, mental health issues, and reduced work productivity [11,12].

Developing strategies to reduce and break up workplace SB, replacing it with activities other than sitting, such as walking or standing, has been the focus of recent studies [13,14]. In particular, the use of digital strategies in desk-based jobs (eg, delivered via mobile phone, activity tracker, or desktop computer) has the potential to provide scheduled prompts, deliver information, give automated tailored feedback, and mediate organizational support and social influences [15]. Recent evidence has highlighted the capability of digital interventions to reduce workplace SB and its associated harmful effects on health and work-related outcomes among office workers [16-18]. However, this research has focused on the traditional office environment, with a dearth of research available on the use of digital interventions to reduce of employees’ SB while working remotely or undertaking hybrid work [19,20].

Multicomponent digital interventions that combine environmental and organizational changes and provision of information and counseling have been reported as more effective than single-component interventions in reducing occupational SB among office workers [16,21]. In addition, the grounding of interventions in a theory of behavior change is key in the success and behavior maintenance of multicomponent interventions [22-24]. Given the limited evidence base on the effectiveness of interventions targeting hybrid work, it is crucial to develop multicomponent digital interventions grounded in sound theory of behavior change to reduce occupational SB among home office workers.

### Objectives

To contribute to a successful implementation of an intervention and, thereby, to its effectiveness, involving stakeholders such as researchers, experts in the addressed topic, and the target population in the development of interventions is necessary to identify needs, priorities, and potential solutions [25]. A previous qualitative study identified which factors influenced employees’ ability to reduce SB when working from home from the employers’ perspective, whereas additional qualitative evidence identified the factors influencing the reduction in SB from the employees’ perspective [26]. This study aimed to go beyond the existing research gaps to incorporate expert opinions to gain a complete picture of all stakeholders and reach consensus on the feasibility of digital work strategies to reduce SB in a home office context. Therefore, the aim of this study was to reach expert consensus on the most feasible and usable digital work strategies as a delivery method to reduce SB in a home office context.

## Methods

### Design

A modified Delphi study was used to elicit a consensus from a spectrum of experts in the field of occupational health. It is a flexible approach combining quantitative and qualitative data. Qualitative data can be collected before, after, or between Delphi rounds [27,28]. This study encompassed 3 survey rounds and subsequent web-based focus group sessions. This study is part of the initial modeling of a multicomponent intervention and of a larger European project (ie, the Click2Move program) aimed at tackling the challenge of occupational sedentarism in hybrid office workers.

This study was based on the recommendations of the Conducting and Reporting Delphi Studies guidelines [29] and the COREQ (Consolidated Criteria for Reporting Qualitative Research) checklist [30].

### Ethical Considerations

This project was approved by the Research Ethics Committee of the University of Vic–Central University of Catalonia (250/2023). This study was conducted in accordance with relevant guidelines and regulations. The invitation email contained general information about the study and a link with further details of the participant information statement, including assurances of anonymity and confidentiality and emphasizing that participation was completely voluntary and participants could withdraw at any time without any consequences. In compliance with ethical research practices, digital informed consent was obtained from all participants before initiating the survey. No incentives were provided to the participants for their involvement.

### Participants and Recruitment

International experts (N=57) identified as leading or established researchers in the field of occupational health (with a specific focus on modification of physical activity [PA] behavior in the workplace), well-being, or computer- or desk-based jobs specialists identified by the research team were invited via email to participate. The experts were selected through a search of published articles that identified highly active authors in the field, whereas the job specialists were identified from professional networks of the involved institutions. The exclusion criterion was not being able to understand and communicate in English.

To maximize participation in the Delphi survey, a reminder email was sent within 1 week. After completing the 3 Delphi rounds, participants who signed the informed consent form for the Delphi study (29/57, 51%) were invited via email to participate in a focus group.

### Procedure

#### Delphi Survey

##### Overview

**Figure 1:**
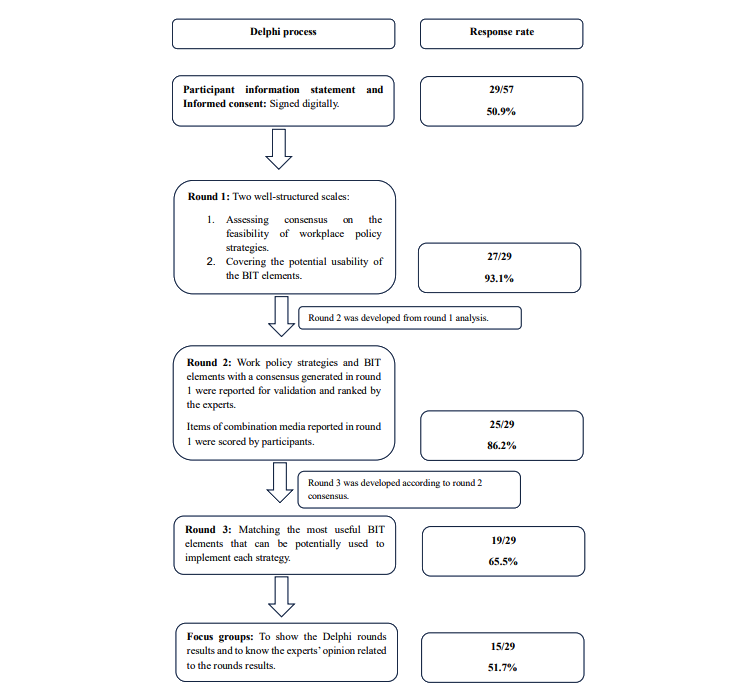
Delphi process. BIT: behavioral intervention technology.

In total, 3 survey rounds were conducted using the LimeSurvey software (LimeSurvey GmbH; Multimedia Appendix 1), which were piloted by 4 members of the research team to ensure the appropriateness of the questionnaire items, wording, and functionality and identify any issues or gaps. No changes in wording were made to the Delphi surveys. If participants agreed to take part, they received an automatic email with the link to the first survey. Participants had 10 days to complete each round of the survey. Individual responses were anonymized, and all participants were invited to complete the second and third rounds of the survey regardless of their participation status in the previous rounds. Attrition was prevented through a short period between rounds and by providing feedback on study findings. A summary of the entire Delphi process is provided in Figure 1.

##### Round 1

Round 1 was carried out between January 13 and 23, 2023. Participants were provided with an explanation of the survey and were asked to provide demographic information (ie, age, gender, and employer organization). Two 9-point Likert scales were used.

The first scale assessed participants’ opinions on the feasibility of work strategies (n=36) for reducing SB when working from home. The work strategies had been selected from a scoping review of the vast majority of real-life PA strategies implemented [31], a systematic review of effective digital interventions to reduce SB [21], and 2 qualitative studies that further explored the perceptions of managers and employees on the factors influencing the ability of employees to reduce their SB in a home office context [26]. The strategies were classified based on the Behavior Change Wheel (ie, environmental planning and service provision, 9/36, 25% of the strategies; regulation, guidelines, and restriction, 15/36, 42% of the strategies; and communication and social support, 12/36, 33% of the strategies) [32].

Participants were invited to score each work strategy. Scores ranging between 1 and 3 suggested a lack of feasibility, scores between 4 and 6 suggested neutral feasibility, and scores between 7 and 9 suggested high feasibility. This scoring was based on the 9-point Grading of Recommendations Assessment, Development, and Evaluation methodology [33]. The feasibility scores considered the 4 dimensions of feasibility: social validity or acceptability, integration into the existing system, practicality, and adaptability (Table 1) [34].

The second scale covered the potential usefulness of the technology-based behavior change elements according to the behavioral intervention technology (BIT) model (ie, messaging and social support [n=6 elements], push notifications [n=5 elements], information delivery [n=9 elements], report and visualization [n=4 elements], digital log [n=3 elements], and passive data collection [n=7 elements]) [35]. Scoring was based on the 9-point Grading of Recommendations Assessment, Development, and Evaluation scale divided into 3 categories: not useful (1-3), neutral (4-6), and highly useful (7-9) [33]. Participants were also invited to provide additional items or observations through an open-ended question for each strategy or BIT element category.

**Table 1:** Feasibility dimensions for the strategies and the related research question for each dimension.

Dimension	Research question
Social validity or acceptability	Is the strategy appropriate, reasonable, fair, and potentially effective for home-based work?
Integration into the existing system	To what extent are the strategies aligned with the infrastructure of home office settings?
Practicality	Can the initiative be implemented using the available resources, time, training, and materials in home-work offices?
Adaptability	Is the initiative flexible enough to fit across the diverse needs of home office work?

##### Round 2

Round 2 started on February 6, 2023, and finished on February 16, 2023. Work strategies from round 1 were ranked based on the *highly feasible* scores for work strategies and *highly useful* scores for BIT elements. A total of 35 work strategies (rather than 36) were ranked in round 2 based on recommendations from round 1 (a similar approach was implemented for work strategies and BIT elements). In this second round, participants were asked to place each work strategy in order of preference considering the most feasible strategies to reduce SB when working from home. The same procedure was followed for the BIT elements. Additional items that appeared in the first survey were scored based on the round 1 approach (ie, the 9-point Likert scale).

##### Round 3

Round 3 was launched on February 17, 2023, and could be answered until February 27, 2023. According to the preferred order of the items ranked in round 2, participants were asked to match the most useful BIT elements that could potentially be used to implement each of the work strategies to reduce SB when working from home. The work strategies included in this round were those with a weight above the median.

#### Focus Groups

To explore in more depth the experts’ opinions and understand their perspectives, focus groups took place.

Focus groups were conducted in English or Spanish by a researcher (AC or IP-S) and a moderator (IP-S, JB-R, or AMS-M) with previous experience facilitating focus groups. The focus groups were held on the web using Microsoft Teams in April 2023. The focus groups lasted a total of 60 minutes. The size of the groups was small (4 participants on average) due to scheduling difficulties and time zone differences among countries.

A guide was developed based on the 3 Delphi rounds (Multimedia Appendix 2). However, the researchers did not follow it in full detail, using the freedom to explore thoughts and ideas based on the responses from each group. First, there was a brief welcome and introduction to explain the aim of the focus group, obtain consent for the recording of the session, and introduce each participant. The results of every Delphi round were then shown. Subsequently, experts were asked about what factors they thought made some strategies more feasible than others considering the 4 dimensions (outlined in Table 1) and what factors may enhance participation among the BIT elements. The last element of the focus group covered the need for any adaptation before the implementation of the strategies in the hybrid work model. The focus groups were recorded, transcribed verbatim, and translated into English if necessary to be analyzed later.

### Data Management and Analysis

Study uptake was calculated as the proportion of participants who accessed the round 1 Delphi survey to that of all potential participants identified. Retention rate referred to the proportion of completed responses for each round and participation in the focus groups. In round 1, responses to the Likert scale in the *highly feasible* or *highly useful* category (score from 7 to 9) were calculated. The median and mean absolute deviation from the median (MAD-M) for each item were also reported to assess the strength and extent of agreement. Medians of 7 to 9 were defined as strong support, medians of 4 to 6 were defined as moderate support, and medians of 1 to 3 were defined as no support. The MAD-M describes the variation in the median values [36]. In round 2, we calculated the weighted average ranking for each item to determine the most highly ranked work strategies and BIT elements for reducing SB among home office workers. The respondents’ most preferred choice had the largest weight, and the least preferred choice had a weight of 1. For achieving consensus, the order-weighted average process was followed through the aggregation of the strategies evaluated in the previous round to be validated in round 2 [37]. Items that achieved consensus were those that were ranked with a weighted average above the median. The additional items that emerged from round 1 were assessed through a 9-point Likert scale in round 2, and the median and MAD-M were calculated. In round 3, the matched items for each work strategy and BIT element were reported, as well as the percentage of respondents.

The focus group analysis was conducted using the principles of reflexive thematic analysis [38,39]. First, a researcher (IPS) familiarized themselves with the data, reading and rereading carefully each transcript to obtain a general impression of the main opinion of the experts and identify repeated concepts. Subsequently, according to the subjectivity of the researcher, an initial open coding process was generated from interesting features and relevant data searching for evidence for themes, namely, patterns of shared meaning underpinned by a central organizing concept. Reflexive thematic analysis was approached in an inductive, semantic, and realistic way. In other words, the construction of codes and themes was directed by the content of the data but not by a topic summary. Codes, concepts, and the core category were recoded and relabeled if necessary after each step until the codes provided the intended depth of the insights. The analytic outputs obtained from the codes and through the active engagement of the researcher with their data were then conceptualized in potential themes and subthemes. Finally, the themes were reviewed, defined, and named. At the end of each step, data were shared with an experienced supervisor (CV-C) for reflecting on how the data were initially coded and consider things that might have been overlooked. MAXQDA 2022 (VERBI GmbH) was used for the analysis.

To ensure a robust analytical process, we considered key aspects of quality and rigor. The first aspect was reflexivity, which involves critical reflection of researcher perspectives and its integration within the analysis and interpretation of the data to develop themes [40]. The study team comprised researchers with expertise in PA and SB in the workplace who have experienced working from home since before or after COVID-19 pandemic. IP-S, who carried out the analysis, is a Spanish physiotherapist and osteopath PhD student who has been working from home for 3 years; CV-C, the supervisor during the analysis, is an experienced nutritionist with a background in health promotion and qualitative research who has been working from home since the COVID-19 pandemic. Second, confirmability, the objectivity of the findings [41], was attained through a critical friend approach whereby an experienced supervisor reviewed interpretations and introduced alternative perspectives, increasing objectivity and confirming the accuracy of the findings. Third, dependability, the reliability and consistency of the study [41], was attained through detailed research procedures (ie, rigorous data collection techniques and well-documented procedures and analyses). Credibility was then built on the reflexivity and triangulation of the data, incorporating more than one researcher in the analysis process [42]. Furthermore, a critical friend approach was used whereby an experienced qualitative researcher, CV-C, critically analyzed the preliminary themes and ensured that the selected quotes provided support for the themes developed. Finally, transferability was enhanced through the transparent and detailed descriptions of the participants and research process [42].

## Results

### Participant Characteristics

A total of 51% (29/57) of the invited experts expressed their interest in participating in the study through the digital informed consent form. Of these 29 participants, 27 (93%) completed the first round, 25 (86%) completed the second round, and 19 (66%) completed the third round. Of those 29 participants, 15 (52%) joined the focus groups.

Most of the first-round Delphi respondents and focus group participants identified as women (16/27, 59% and 8/15, 53%, respectively), with 48% (13/27) of first-round respondents aged between 36 and 45 years.

A wide variety of countries across Europe were represented. However, non-European countries such as Australia (5/29, 17% of the participants) were also represented.

### Delphi Survey

#### Feasibility of Work Strategies

##### Round 1

Multimedia Appendix 3 shows the results of round 1, which considered the 36 work strategies divided into 3 categories. A total of 69% (25/36) of the work strategies were ranked as highly feasible.

In total, 7 environmental planning and service provision work strategies were rated as highly feasible with strong support. Activity trackers (20/27, 74%; median 8, MAD-M 1.41); workstation accessories (19/27, 70%; median 8, MAD-M 1.59); and relocation of home office supplies such as the bin, printer, or water source (19/27, 70%; median 8, MAD-M 1.30) were the 3 work strategies considered as highly feasible for reducing SB when working from home. Active workstations equipped with treadmills were reported to be unfeasible (19/27, 70%; median 2, MAD-M 1.48).

In total, 53% (8/15) of the work strategies regarding guidelines, regulations, and restrictions were rated as highly feasible with strong support. Self-monitoring of sedentary and activity behaviors (20/27, 74%; median 8, MAD-M 1.30), substitution of sedentary tasks (eg, reading, answering a phone call, or participating in a videoconference) with standing or incidental movements (20/27, 74%; median 7, MAD-M 1.37), and creating an action plan (20/27, 74%; median 7, MAD-M 1.11) were the most highly useful. The least feasible strategy was the locking or blocking of the screen or keyboard for specific durations (11/26, 42%; median 5, MAD-M 2.19).

Regarding the communication and social support work strategies, feedback on activity progress and achievement of goals (22/27, 81%; median 7, MAD-M 1.74); providing information to increase awareness and knowledge (20/27, 74%; median 8, MAD-M 1.37); and the provision of awards, rewards, or incentives by the company to achieve goals (19/27, 70%; median 7, MAD-M 1) were ranked as the most feasible for reducing SB when working from home. Most of the work strategies included in this category (10/12, 83%) were rated by the respondents as highly feasible, and none of them were considered unfeasible. No new items emerged from open-ended questions.

##### Round 2

The ranking list of the work strategies with the weighted average (range 14.6-0.48) is reported in Table 2. Most items reported as highly feasible when working from home in round 1 (18/25, 72%) were scored above the median (3.64) in round 2, with the exception of self-selected reminders (3.24); active breaks such as stretching, walking, or strengthening exercises (2.36); real-time records (2.32); high or height-adjustable chairs (1.96); exercise accessories (1.52); and social networking for sharing experiences (0.48).

**Table 2:** Ranking of highly feasible work strategies (results of round 2).

Strategy	Weighted average score
Feedback on activity progress and goal achievement	14.6
Create an action plan—increase standing breaks or replace sitting time, how long, how often, when, and how (eg, when the phone rings)	13.04
Standing while reading, answering phone calls, or conducting videoconferences	13.04
Providing information to increase awareness and knowledge of the dangers associated with prolonged sedentary behavior and the potential benefits of reducing it or breaking it up	12.88
Self-monitoring sedentary and activity behaviors (ie, activity tracker or a diary log)	10.76
Short breaks (5-10 min) approximately every 60 min of sitting time	10.36
Scheduling (blocking) 5-10–min breaks between meetings on the calendar	8.6
Information and support about the strategies and goals and reminders	8.04
Setting tailored goals for reducing sitting time	7.24
Activity demonstrations to perform during the breaks	7.12
Height-adjustable desks or desk platforms	6.92
Relocation of home office supplies (eg, bins and printers)	6.8
Incidental moving while reading, answering phone calls, or conducting videoconferences	5.76
Awards, rewards, or incentives to achieve goals or recommendations	5.48
Standing desks	4.6
Point-of-choice or point-of-decision prompts	4.4
Workstation accessories (seated footrests, standing footrests, or sit-stand antifatigue mats for standing desks)	4.24
Motivational messages from managers	3.64
Wellness coaches supporting the employees during breaks	3.48
Self-selected reminders to achieve goals	3.24
Blocking the screen or keyboard for breaking up sitting time unless conducting a meeting	3.24
Active workstation with underdesk stepper or pedaling device	2.52
Short breaks (5-10 min) approximately every 30 min of sitting time	2.48
Team or individual activity challenges	2.4
Active breaks, such as stretching, walking, or performing strengthening exercises for ≥10 min	2.36
Scheduling (blocking) snack breaks on the calendar	2.32
Real-time records or feedback	2.32
Active lunchtime, such as Pilates, yoga, walking, or cycling	2.04
High chairs or height-adjustable chairs	1.96
Social comparison	1.68
Exercise accessories, such as rubber bands, wooden sticks, or mats	1.52
Active workstation equipped with treadmill	1.44
Short breaks (5-10 min) approximately every 40 min of sitting time	1.36
Competition among peers	0.92
Social networking for sharing experiences	0.48

#### Usefulness of BIT Elements

##### Round 1

Multimedia Appendix 4 shows the results of round 1 considering the usefulness of the BIT elements divided into 5 categories.

In total, 33% (2/6) of the BIT elements from the messaging and social support category were ranked as highly useful with strong support: social challenge features (20/27, 74%; median 7, MAD-M 1.22) and gamification (14/26, 54%; median 7, MAD-M 1.78). None of the messaging and social support items were considered not useful. Smartphone app and desktop application interfaces were identified to be the 2 most useful BIT elements within the push notification category (12/27, 44%; median 7; and MAD-M 1.41 for smartphone app interface and 15/27, 56%; median 7; and MAD-M 1.18 for desktop application interface). The other 3 push notification elements were ranked as highly useful or neutral with medians between 5 and 6, indicating moderate support. A combination of different media was the preferred item in the delivery of information category (14/24, 58%; median 7, MAD-M 1.71), with the other elements within the category receiving moderate support and being considered neutrally or highly useful. A total of 15 new combined digital items, such as videos and images, moving images on websites, or reminders combined with social challenges, emerged from open-ended responses to the combination of media element in the information delivery category. Blending was considered more useful than isolated media. Real-time data (19/27, 70%; median 7, MAD-M 1.48) and data summaries via the app interface (14/27, 52%; median 7, MAD-M 1.67) were considered the most useful in the report and visualization category. The 2 remaining items (data summaries via email or SMS text message and websites) were ranked as neutral. The 3 most useful BIT elements to track activity were wrist-based activity trackers (17/26, 65%; median 7, MAD-M 1.69) and using a mobile phone diary (17/27, 63%; median 7, MAD-M 1.41) or smartphone sensors (13/26, 50%; median 7, MAD-M 1.61). The only element considered as not useful to track activity was waist-based activity trackers (10/26, 38%; median 5, MAD-M 1.85).

##### Round 2

Overall, BIT elements reported as highly useful to support work strategies were ranked at the top in each category (Table 3). In the messaging and social support category, items ranked above the median score (2.52) coincided with highly useful items in round 1 (ie, social challenge features and gamification), but chats (eg, WhatsApp groups) were added to the top of the ranking. SMS text messages were added to the 2 BIT elements in the push notification category considered as highly useful in round 1, which were scored above the median (2.00) in round 2. In addition to combination of media, videos, interfaces in smartphone apps and desktop applications (same as in the push notification category), images, and websites were among the items scored above the median (3.24) in the information delivery category. The new items that emerged from the open-ended responses in round 1, specifying potential combinations of media for information delivery described as highly useful, were a combination of mobile phone apps and desktop applications; a combination of personalized messages, reminders, and social challenges; a combination of videos, text, and images within an interactive website; and a combination of a mobile app with social media (Table 4). In the report and visualization category, the 2 items ranked as highly useful in round 1 remained above the median (2.32) in round 2. The median of the BIT elements to track activity was 3.56. In round 2, a total of 2 more items were added as highly useful to the 3 identified in round 1: leg-based activity tracker and computer software. In total, 17 BIT elements were considered highly useful in round 2.

**Table 3:** Ranking of highly usable behavioral intervention technology (BIT) elements (results of round 2).

BIT element to communicate information			Weighted average scores (SD)
**Messaging and social support**			
	Social challenge features (cooperative activities, eg, reach 10,000 steps among all members of the department)	5.16	
	Gamification features (competition among colleagues)	4.48	
	Chats (eg, WhatsApp groups)	3.28	
	Emails	1.76	
	Calls	1.4	
	Forums	1.64	
**Push notifications**			
	App interface—text and sound and vibration in smartphones	4	
	Application interface—computer screen notification in desktop computers	3.08	
	SMS text messages	2	
	Chats	1.64	
	Emails	1.52	
**Information delivery**			
	Combination of media	7.6	
	Videos	6	
	App interface—smartphones	4.44	
	Images	4.36	
	Application interface—desktops	3.24	
	Websites	3.24	
	Emails	3.16	
	SMS text messages	2.36	
	Audios	1.44	
**Report and visualization**			
	Real-time data via app interface	2.96	
	Data summary via app interface	2.84	
	Data summary via email or SMS text message	1.8	
	Data summary via website	1.24	
**BIT elements to track activity**			
	Mobile phone diary (manual entry)	7.72	
	Web-based questionnaire (manual entry)	3.52	
	Computer software diary (manual entry)	1.6	
**Passive data collection**			
	Wrist-based activity tracker	8.6	
	Leg-based activity tracker	3.64	
	Smartphone sensors	3.6	
	Computer software	3.6	
	Waist-based activity tracker	3.28	
	Electronic workstation	3.08	
	Cushions on chairs	2.04	

**Table 4:** Results for the new items from round 1 scored in round 2—behavioral intervention technology (BIT) elements.

New BIT element	Level of usefulness, n (%)												Median (MAD-M^a^)
	Not useful				Neutrally useful				Highly useful				
	Score of 1	Score of 2	Score of 3	Score of 4		Score of 5	Score of 6	Score of 7		Score of 8	Score of 9		
Mixture of videos, information, and images within an interactive website (n=23)	0 (0)	1 (4)	2 (9)	2 (9)		0 (0)	6 (26)	5 (22)		6 (26)	1 (4)	6 (1.48)	
App and social media (n=23)	0 (0)	1 (4)	3 (13)	0 (0)		2 (9)	1 (4)	10 (43)		4 (17)	2 (9)	7 (1.30)	
Videos and images (n=23)	0 (0)	1 (4)	1 (4)	1 (4)		4 (17)	3 (13)	12 (52)		1 (4)	0 (0)	6 (1.17)	
Audio and video combination with an explanation (educational purposes; n=23)	0 (0)	0 (0)	2 (9)	1 (4)		4 (17)	6 (26)	2 (9)		3 (13)	3 (13)	6 (1.48)	
Moving images on a website (n=22)	1 (5)	1 (5)	3 (14)	2 (9)		4 (18)	7 (32)	3 (14)		1 (5)	0 (0)	5 (1.41)	
Smartphone and desktop with website or app for background information (n=22)	1 (5)	0 (0)	1 (5)	0 (0)		3 (14)	3 (14)	6 (27)		7 (32)	1 (5)	6.5 (1.39)	
Personalized messages and app (n=22)	0 (0)	0 (0)	0 (0)	0 (0)		1 (5)	3 (14)	8 (36)		5 (23)	5 (23)	7 (0.91)	
Reminders combined with an app (n=22)	0 (0)	0 (0)	2 (9)	0 (0)		1 (5)	4 (18)	4 (18)		8 (36)	3 (14)	7 (1.27)	
Reminders combined with social challenges (n=21)	0 (0)	0 (0)	1 (5)	0 (0)		2 (10)	3 (14)	4 (19)		6 (29)	5 (24)	7 (1.29)	
Forum for general information and smartphone messages (eg, SMS text messages) for specific tailored information (n=21)	1 (5)	1 (5)	0 (0)	5 (24)		2 (10)	6 (29)	2 (10)		3 (14)	1 (5)	5 (1.67)	
Forum for general information and app for specific tailored information (n=21)	1 (5)	1 (5)	1 (5)	1 (5)		4 (19)	6 (29)	1 (5)		4 (19)	2 (10)	5.5 (1.67)	
Forum for general information and email for specific tailored information (n=21)	2 (10)	1 (5)	2 (10)	2 (10)		3 (14)	5 (24)	4 (19)		2 (10)	0 (0)	5 (1.71)	
Website for general information and smartphone messages (eg, SMS text messages) for specific tailored information (n=23)	0 (0)	1 (4)	3 (13)	4 (17)		3 (13)	5 (22)	4 (17)		3 (13)	0 (0)	4.5 (1.65)	
Website for general information and email for specific tailored information (n=23)	0 (0)	2 (9)	2 (9)	4 (17)		5 (22)	5 (22)	3 (13)		1 (4)	1 (4)	4.5 (1.52)	
Website for general information and app for specific tailored information (n=23)	0 (0)	1 (4)	2 (9)	1 (4)		6 (26)	6 (26)	3 (13)		3 (13)	1 (4)	5 (1.43)	

^a^MAD-M: mean absolute deviation from the median.

#### Round 3

After the description of the priorities of the expert panel, the BIT elements that were considered most suitable for implementing each of the work strategies are shown in Multimedia Appendix 5, including the 18 highly feasible work strategies and 16 rather than 17 highly useful BIT elements because social challenge features were considered within the gamification features in round 3.

The use of wrist-based activity trackers was identified as useful by 63% (12/19) of respondents for self-monitoring sedentary and activity behaviors. Using a computer software was considered useful to schedule 5- to 10-minute breaks between meetings (9/19, 47%), whereas a desktop application interface was considered useful to support strategies such as short breaks every 60 minutes of sitting time (6/19, 32%). Videos were described to be useful when delivering activity demonstrations to perform during the sitting breaks (9/19, 47%). Other BIT elements identified were gamification features, such as social challenges, which are used for providing awards and rewards and offering incentives (8/19, 42%). However, setting tailored goals (8/19, 42%); creating an action plan (6/19, 32%); standing while reading, answering calls, or conducting videoconferences (6/19, 32%); providing feedback on activity progress (4/19, 21%); and motivational messages (4/19, 21%) were mainly recognized to be best delivered through a smartphone app. Providing information and support was considered to be best delivered through a combination of media or a smartphone app (5/19, 26% each). Combination of media was also chosen by most of the respondents as the most useful mode for providing information to increase awareness and knowledge (5/19, 26%) and for promoting incidental movement (3/19, 16%). Environmental strategies such as height-adjustable desks (11/19, 58%), relocation of home office supplies (5/19, 26%), standing desks (9/19, 47%), and workstation accessories (9/19, 47%) did not correspond with any BIT elements according to most of the respondents. For point-of-choice or point-of-decision prompts, there were no corresponding BIT elements to support their implementation according to most of the respondents for this strategy (4/19, 21% of the sample), although 16% (3/19) considered that this strategy could be delivered via computer software.

### Focus Groups

#### Overview

A total of 2 indicative themes were generated from the analysis of the focus groups regarding the experts’ perceptions in relation to the Delphi results on the potential feasibility and usefulness of strategies and digital elements to reduce SB in a home office context. The first theme referred to the integration of the digital strategies for reducing SB when working from home, which included 3 subthemes: social influences, physical environment, and individual factors. The second theme was related to the adaptability of the strategies to reduce SB in the office and home context.

#### Theme 1: Integration of the Digital Strategies for Reducing SB When Working From Home

##### Overview

This theme involves the importance of combining multiple levels of strategies to facilitate and maximize the strategies’ implementation and their effectiveness in reducing SB when working from home. According to the experts, while organizational-level factors such as organizational support or policy changes may not be immediately feasible on their own, they are essential for achieving sustainable change. The same was considered true for information provision—it alone is not enough to motivate behavior change, although it is an important factor at the individual level to increase individuals’ knowledge. In addition, environmental changes are required to promote less sitting and more movement during working hours. Therefore, experts highlighted that the following implementation levels need to work in tandem to achieve sustainable behavior change.

##### Social Support

Experts explained the need for the company to support the culture of active and healthy workplaces to motivate the employees’ behavior change. Social support may be achieved by aligning the company policies and culture with the support of the manager (eg, motivational messages) to be able to increase the integration of digital strategies for reducing SB when working from home:

[I]t is based on what the management or the philosophy of the company or the company policy wants to refer to their workers. If those at the top are not proactive in...the chain, it will decrease.

E13; focus group 4; paragraph 43

While the target behavior is contextualized within the domain of working from home, participants identified interaction with colleagues as a relevant factor to motivate employees toward behavior change. For example, social modeling may motivate home office workers to reduce their SB by sharing activities around the sitting time breaks. However, social interaction can be perceived negatively if a competition component is present in the strategies. This may be due to competing against people whom they do not know or due to the bottom position of some workers during the challenges. Therefore, cooperative challenges may be the solution, in which every worker contributes to the challenge without a competition element:

[W]hen people find themselves in an environment where they’ve got to adapt behaviourally, seeing what can be done and observing how others might do it, can be quite powerful...we are social animals, monkey see, monkey do and that social modelling element to me jumps out as really quite important and not to be lost.

E01; focus group 1; paragraph 44

##### Physical Environment

The home office context was seen by some participants as a limiting factor in engaging in sufficient PA during work hours due to the reduced space compared to the office context. For example, having (normally) no more than 1 floor at home, implying the use of stairs, or the lack of large spaces for walking are barriers to be active while working at home. Although applying physical changes such as standing desks may be a solution to break up sitting time at work, it appears to be a less feasible strategy in the home office context due to the expensive cost for the companies:

I’m a big advocate of standing desks, standing workstations in terms of being effective but I appreciate that in this situation it’s probably not feasible for them to be incorporated in the home-office.

E10; focus group 3; paragraph 7

Nevertheless, experts highlighted the importance of combining these environmental changes with prompts or reminders for the success of the behavior change integration when working from home. Regarding point-of-decision prompts, experts highlighted that mobile phone notifications were more feasible for reducing SB than desktop notifications because they cause less disturbance:

[W]hen we moved into our new offices a few years ago, we all got standing desks and just observing...I don’t see anybody using. So, if there’s something for me in there about prompting/reminders, do you know?... So, I think the prompting is really important.

E011; focus group 3; paragraph 11

##### Individual Factors

Experts mentioned the difference in literacy between *PA* and *sitting time*, believing that workers may understand the term *PA* more easily than the term *sitting time*. This may be due to the lack of knowledge on the importance of reducing sitting time behaviors, which could be perceived as a barrier when only reporting sitting time feedback. Therefore, educational material or information, one of the highlighted strategies to be used together with social support and physical environment strategies to achieve SB reduction when working from home, delivered through a combination of media (ie, smartphone, desktop, and websites), was considered the most adaptative medium to increase the employees’ knowledge. Experts emphasized that the information delivered must be simple and short, especially with the use of videos:

I think the combination of media is probably the way forward because I guess different organisations will also have different ways of rolling out information. I know a lot of companies nowadays that they all have their own company app, and they do a lot of communication via their app that all their employees have access to.

E10; focus group 3; paragraph 42

Furthermore, feedback on the respondents’ own behavior was identified as a key strategy to motivate individuals and achieve behavior change. The best frequency at which to deliver feedback could be in real time as this adapts to each individual’s schedule. However, a daily summary feedback approach was also considered helpful in combination with real-time feedback:

For the summary—daily maybe? But I probably go for the combination of the two, so you probably have some sort of summary data to share with them but still, it’s nice to see at the moment what you did.

E05; focus group 2; paragraph 67

#### Theme 2: Adaptation to the Home Office Environment

This theme refers to the flexibility of the strategies to fit the diverse needs of hybrid work. All the experts mentioned the capacity of the strategies to be adapted to all environments without the need to be changed to fit in the home or office context:

I think you want to have the same or approach or whatever you use for say, remote working, I would also use for hybrid working because I guess a lot of strategies are going to be the same...So, I think you could maybe just replicate or duplicate what you’re doing here for the hybrid workers.

E10; focus group 3; paragraph 64

However, some strategies need to be adapted to each individual depending on the type of work they perform. For example, walking while on a call may be a good strategy for a call center, but it might not work in another type of job. Therefore, it is valuable to provide different strategies to allow everyone to choose their preferred one to change their behavior:

[D]ifferent populations need a different approach, right? So very much dependent on the type of work that people are doing, you’re going to have to approach them differently. So, the task set is going to help determine what options you actually have to still do your work but would be doing it sitting down all the time.

E05; focus group 2; paragraph 29

Therefore, experts highlighted the importance of providing a list of strategies that can be adapted to employees’ needs for reducing sitting and moving more during their working hours:

So instead, you look towards providing a menu of strategies that you know are effective or have at least been shown to be feasible and acceptable and not shown to have any harm and you know, have benefits maybe in terms of cultural elements of it and that a lot of those would be applicable for the home environment but would also be applicable obviously for the workplace environment.

E02; focus group 1; paragraph 65

#### Summary

In general, no dissonance was found among the responses of the different experts. Strategies such as feedback, social modeling, breaks every hour, prompts via mobile phone every hour, educational material, and organizational support (eg, motivational messages) were the most discussed and considered the most feasible. Other strategies such as physical changes (ie, height-adjustable desks) were considered unfeasible despite being ranked higher in the Delphi rounds. Finally, although certain strategies such as the creation of an action plan, standing while reading and answering calls, blocking 5 to 10 minutes between meetings, or setting tailored goals for reducing sitting time obtained high scores in the Delphi rounds, these topics were discussed less among the experts during the focus groups.

## Discussion

### Principal Findings

This study achieved international expert consensus on 18 potentially feasible work strategies to reduce home office SB delivered through 16 potentially useful digital elements. These results seem to be transferable to both office and home settings based on experts’ responses. Moreover, the experts highlighted the importance of multiple factors (ie, social, individual, and environmental) working in tandem to facilitate and maximize the strategies’ implementation, as well as to achieve behavior change.

### Comparison With Prior Work

Wrist-based activity trackers were identified by most of the experts (12/19, 63%) as useful for self-monitoring activity and SB. Previous research has highlighted the feasibility and usability of these devices for self-monitoring SB as well as workers’ acceptability of these approaches in the office context [43,44]. In addition, these strategies have demonstrated effectiveness in reducing SB in such settings [44]. Some studies have highlighted limitations in recognition of postures and step detection when using wrist-based devices compared to leg-based devices [45-48]. However, few experts considered leg-based activity trackers for self-monitoring activity and SB due to the discomfort they may cause. This suggests that future interventions incorporating activity trackers with the aim of reducing SB should first validate the devices for their subsequent implementation and effectiveness evaluation in home office settings.

In the literature, computer visual feedback on activity patterns is considered to be an enabling factor for increasing motivation and awareness among office workers [49]. However, experts preferred smartphone apps as they are able to provide real-time feedback on activity progress. In addition, feedback may be an indication of progression toward a defined goal [43]. In this study, experts stated that smartphone apps could be a useful tool for setting tailored goals. Evidence has demonstrated the effectiveness of smartphone apps in reducing SB and promoting PA in office settings [16]. Hence, smartphone apps may be a potential mode of delivery for setting tailored goals and providing feedback on the move, which should be evaluated in future home office environment interventions.

Previous research has demonstrated that short activity breaks (ie, walking) every 30 minutes result in reductions in office SB time [50] but also in reductions in fatigue and improvements in energy and mood [51]. However, in this study, experts considered breaks every 60 minutes more feasible. According to recent literature, a possible explanation may be related to the fact that workload and time pressure were identified by employees as the most common barriers for reducing and breaking up SB [52], making it difficult to stop more frequently. Moreover, replacing SB with active working tasks (eg, incidental moving while reading and answering calls) may overcome these barriers [49]. Working remotely increases opportunities to perform active tasks or even other light-intensity PA that may increase the potential benefits of reducing SB and its harmful health effects among office workers [9,10,53]. Experts reflected on the importance of providing clear instructions and modeling through video demonstrations as a key digital element to enhance active breaks among employees when working from home. This may have implications for future studies evaluating the most effective active break duration through video demonstrations and the implications of these breaks regarding SB time and its health effects in home office contexts.

On the basis of experts’ responses, there is a literacy gap between the terms *PA* and *SB*, with activity trackers largely focused on PA instead of SB [52]. Therefore, educational material may be a key aspect to increase knowledge for behavior change and reduce the lack of understanding of SB and its subsequent health implications, which was highlighted by experts. Educational material is an essential and effective strategy for workers to increase awareness of SB’s health consequences [52,54]. In our findings, a combination of media was preferred to deliver information to increase awareness and knowledge. Therefore, it is recommended that future studies include educational material to increase knowledge and awareness among home office workers.

Despite the relevance of knowledge and awareness to achieve behavior change, social and organizational support is a factor noted by workers in the current literature for facilitating and encouraging movement in the workplace [55-57], as well as by managers in the home office setting [26]. Home office work is more related to negative emotions than office work due to the isolation and loneliness; therefore, social support is even more important in this context [58]. Gamification features, including social challenges, were identified as a useful digital element to deliver strategies such as awards, rewards, or incentives to achieve goals or recommendations for reducing SB among home office workers. This strategy could provide social support and motivation. Motivational messages from managers are also an organizational support strategy, which experts suggest could be delivered through smartphone apps. Thus, future studies should evaluate the acceptability of these social and organizational strategies among both managers and employees, as well as their effectiveness in the home office context.

Environmental work strategies such as standing desks, height-adjustable desks, or workstation accessories were mostly not matched with any digital element. Moreover, experts recognized work strategies such as height-adjustable desks as unfeasible in the home office environment, primarily due to the associated costs. Recent evidence has demonstrated the high cost of such desks (eg, standing desks or height-adjustable desks) compared with nondesk interventions [59]. Workstation accessories (eg, desk platforms) or relocation of office supplies are less expensive strategies [60] that were deemed more feasible and usable by experts and may be implemented in home office settings. Therefore, home office interventions may include environmental work strategies in a similar way to office interventions but with a lower associated cost.

On the basis of the findings presented in this paper, physical environmental strategies need to work in tandem with social support and individual strategies to achieve behavior change. A recent systematic review showed that theory-based interventions, a relevant step in multicomponent intervention development [25], that include social, physical, and individual elements are more effective than non–theory-based interventions [21]. Furthermore, stakeholder engagement (eg, consultation or coproduction among researchers, experts on the topic, and the target population) should be encouraged throughout the development process [25]. Recent studies have highlighted the need for a top-down approach whereby managers should provide support to employers [26]; hence, the implementation of strategies should be an organizational initiative. Although evaluating effectiveness on outcomes has traditionally been the primary focus of interventions in this discipline, in particular to encourage their adoption in the real world, the feasibility, acceptability, cost-effectiveness, and scalability of interventions is increasingly important to ensure the translation of findings from research to practice [25,61]. This study provides new insights for future research on the design of home office interventions aimed at reducing SB based on the learned experiences of occupational health experts. However, we were not able to cover the entire audience’s perspective, so new qualitative studies exploring the perspectives of different stakeholders (eg, end users) may further inform about the feasibility and usability of digital workplace strategies.

In addition, experts considered that no adaptations would be needed to transfer these home office strategies to hybrid work, the current modus operandi for many desk-based workers. However, the experts who participated in this study were mainly from high-income countries; therefore, such perceptions may vary among people from other regions. Hybrid work presents opportunities for promoting PA by providing flexibility to integrate occupational, leisure, lifestyle, and incidental activities [9] but also for improving health and well-being [62]. Hence, the results of this study may be transferable to hybrid work in high-income countries, whereas hybrid work should receive greater consideration in future interventions given cultural variability.

### Strengths and Limitations

The main strength of this study is the novelty of the home office context to deliver strategies for reducing SB through digital elements. The fact that focus groups were conducted after a Delphi study enabled us to elicit additional views and opinions on the Delphi results from the experts’ perspectives, consolidating the consensus. Nevertheless, we did not share the analytical categories, interpretations, or conclusions from the thematic analysis with the experts, which may affect the analysis’s credibility. The entire study was conducted in a rigorous manner, with high participant response rates and representation from international experts. However, some strategies may have been overlooked due to the nonsystematic search approaches used, and most of the international experts were from high-income countries, limiting the applicability of these findings to those communities. Some other limitations were present in the study. First, despite the rigor in the methodology used to engage experts, not all responses were complete, meaning that data were sometimes missing from some questions. Second, as is the case in any Delphi survey, the data gathered were based on subjective opinion and the expertise of the participants. Third, experts acted as representatives of the stakeholders; thus, the inclusion of other stakeholders such as end users would also have been important to address other, more diverse perspectives.

### Conclusions

This study presents the consensus of international experts on feasible work strategies and useful digital elements as a mode of delivery of these work strategies to support the reduction in SB in home office or hybrid contexts. Consensus was achieved on 18 work strategies and 16 BIT elements. Future interventions should implement and evaluate the effectiveness of these strategies but also their feasibility and acceptability in home office settings.

## Appendix

Multimedia Appendix 1 Delphi surveys. The document shows the completed survey of round 1 of the Delphi study on the first page. On the second page, the file presents the completed survey of round 2, and the third page shows the completed survey of round 3.

Multimedia Appendix 2 Focus group guide. The file shows the complete guide with the times of the focus groups.

Multimedia Appendix 3 Feasibility of the work strategies (results of round 1).

Multimedia Appendix 4 Usefulness of behavioral intervention technology elements (results of round 1).

Multimedia Appendix 5 Matching behavioral intervention technology elements with work strategies on which consensus was achieved (round 3 results).

## Abbreviations

BIT: behavioral intervention technology
COREQ: Consolidated Criteria for Reporting Qualitative Research
MAD-M: mean absolute deviation from the median
PA: physical activity
SB: sedentary behavior
