# Supplementary material for: Development of Digital Strategies for Reducing Sedentary Behavior in a Hybrid Office Environment: Modified Delphi Study
Source: JMIR Hum Factors. 2025 Apr 8;12:e59405. doi: 10.2196/59405 (PMC12015347; doi:10.2196/59405)
Supplement: Multimedia Appendix 2 [file humanfactors_v12i1e59405_app2.docx]

**Focus group of the Delphi study**

**Introduction – 5 min**

- Welcome and thank you: “We are grateful for your willingness to share your expertise with us. Your insights and perspectives will be instrumental in shaping the outcomes of our study.”
- Introduction of the interviewer: Alan Coffey/Iris Parés
- Aim of the interview: To present the results of the Delphi study and to explore the reasons of prioritization of the strategies and behavioural intervention technology (BIT) elements to reduce sitting time while working from home.
- Session content: Show the results of Delphi study through the ppt and invite them to provide their opinions according to the main reasons for the prioritization for the strategies considering the feasibility in the following dimensions:
  - Social validity or Acceptability: Is the strategy appropriate, reasonable, fair and potentially effective for home-based work?
  - Integration into the existing system: To what extent are the strategies aligned with the infrastructure of home-office settings?
  - Practicality: Can the strategy be implemented with the available resources, time, training, and materials in home-work offices?
  - Adaptability: Is the strategy flexible enough to fit across the diverse needs of home-office work?

In addition, discuss the prioritization of the technology elements according to their usage for implementing the strategies.

- Approximate duration: 60 minutes
- Recording: consent

**Focus group**

1. Short expert’s introduction: name and functions
2. Strategies:
   1. Strategies were divided into 3 groups (environmental planning and service provision; guidelines, regulations, and restrictions; communication and social support). **3 min**

Show the results: the prioritized strategies are shown in the 4^th^ and 5^th^ slide of ppt. Explain them that the blue strategies are environmental planning and service provision, the red strategies are guidelines, regulations, and restrictions, and the green colour are communication and social support strategies. The left site strategies are the most feasible according to their ranking. In the 5^th^ slide there are the less feasible strategies. The most feasible strategies according to the results are green and red strategies.

- 1. Questions: **15 min**
     1. What factors do you think made the priorization of some strategies more feasible than others for home-office settings? Discuss the results considering the acceptability, integration into the existing system, practicality, and adaptability dimensions.
     2. Considering that the hybrid format is the most common nowadays, do you think the higher ranked strategies may be also transferred to the in-site office settings? If so, do they need any adaptation?

1. BIT elements: **20 min**
   1. BIT elements were divided into two big groups (to communicate information and to track activity), the first group was divided into four small groups (messaging and social support; notification push; information delivery; report and visualisation), the second big group was divided into two small groups (digital log (manual entry), and passive data collection). The higher score indicates greater usability.
   2. Messaging and social support: the prioritized items were (8^th^ slide ppt).
      1. Questions:
         1. What factors do you think made some elements more useful to generate social support than others? Discuss the results.
         2. How do you think these BIT elements can be applied to the home-office context?
   3. Notification push: the prioritized items were (9^th^ slide ppt).
      1. Questions:
         1. What factors do you think determined these elements are the most useful for notification push? Discuss the results.
         2. Do you think that a combination of smartphones and desk-based notifications could more useful than one or the other?
   4. Information delivery: the prioritized items were (10^th^ slide ppt). The relevance of using a combination of different media such as videos, text, and images within an interactive app for delivery information to the employees was highlighted in the Delphi-study.
      1. Questions:
         1. Which combination of media do you think could be the most useful to deliver information such as information on strategies to reduce sitting time, benefits of reducing sitting time, …?
   5. Report and visualisation: the two more scored items were (11^th^ slide ppt).
      1. Questions:
         1. What factors do you think determined real-time data as an essential BIT element? In which frequency should real-time data be provided? live, every 5 minutes/10 minutes/15 minutes, …? Frequency of reports: Daily, weekly, …? With reasons.
   6. Track activity: the five highest scored items were (12^th^ slide ppt)
      1. Questions:
         1. What factors made you think a wrist-based device may be useful for tracking and providing feedback about home-office workers?
         2. What information may a mobile phone diary add?
2. Matching BIT elements with work strategies: **10 min**
   1. Results: Creating an action plan, setting tailored goals, reporting feedback on activity progress and goal achievement, providing educational materials, and inform about strategies for breaking and reducing sitting time, were mainly recognised to be performed through a mobile phone application. Using a desktop application was also considered useful to provide point of decision prompts and reminders, as well as to support strategies such as schedule short breaks between meetings from the calendar or every hour without movement. 63% of respondents identified wrist-based tracker as the best BIT element to self-monitoring sedentary and activity behaviours.

Other BIT elements identified were gamification features including cooperative challenges, rankings, etc., for rewarding and offer incentives. A combination of media was also preferred for providing educational information and materials, and to inform and support strategies. The use of videos was described to be useful when delivering motivational messages and activity demonstrations to perform during the sitting breaks.

- - 1. Question:
       1. Considering the big proportion of hybrid work nowadays, which combines home-office work and office work, and given that this Delphi study is related to home-office work, do you think some adaptation is needed to implement digital solutions based on home-office work for reducing sitting time in the office environment?
       2. Would you add or comment anything based on these results?

**Wrap-up and thank you –** **5 min**
