# Supplementary material for: Development of Digital Strategies for Reducing Sedentary Behavior in a Hybrid Office Environment: Modified Delphi Study
Source: JMIR Hum Factors. 2025 Apr 8;12:e59405. doi: 10.2196/59405 (PMC12015347; doi:10.2196/59405)
Supplement: Multimedia Appendix 3 [file humanfactors_v12i1e59405_app3.docx]

**Multimedia Appendix 3.** Feasibility of the work strategies (results of round 1).

| Work policy strategy | | Level of feasibility, n (%) | | | | | | | | | Median (MAD-M^a^) |
| --- | --- | --- | --- | --- | --- | --- | --- | --- | --- | --- | --- |
|  | | Unfeasible | | | Neutrally feasible | | | Highly feasible | | |  |
|  | | Score of 1 | Score of 2 | Score of 3 | Score of 4 | Score of 5 | Score of 6 | Score of 7 | Score of 8 | Score of 9 |  |
|  | | | | | | | | | | | |
| **Environmental planning and service provision** | | | | | | | | | | | |
|  | Activity tracker (n=27) | 0 (0) | 1 (4) | 0 (0) | 3 (11) | 2 (7) | 1 (4) | 6 (22) | 8 (30) | 6 (22) | 8 (1.41) |
|  | Workstation accessories (seated footrests, standing footrests, or sit-stand antifatigue mats; n=27) | 1 (4) | 1 (4) | 1 (4) | 1 (4) | 2 (7) | 2 (7) | 4 (15) | 8 (30) | 7 (26) | 8 (1.59) |
|  | Relocation of home office supplies (eg, bins and printers; n=27) | 1 (4) | 2 (7) | 0 (0) | 0 (0) | 4 (15) | 1 (4) | 5 (19) | 3 (11) | 11 (41) | 8 (1.81) |
|  | Height-adjustable desks or desk platforms (n=27) | 1 (4) | 1 (4) | 2 (7) | 1 (4) | 0 (0) | 4 (15) | 5 (19) | 9 (33) | 4 (15) | 7 (1.59) |
|  | High chairs or height-adjustable chairs (n=26) | 1 (4) | 1 (4) | 1 (4) | 0 (0) | 1 (4) | 7 (27) | 5 (19) | 6 (23) | 4 (15) | 7 (1.46) |
|  | Exercise accessories, such as rubber bands, wooden sticks, or mats (n=27) | 1 (4) | 1 (4) | 3 (11) | 2 (7) | 2 (7) | 3 (11) | 6 (22) | 6 (22) | 3 (11) | 7 (1.78) |
|  | Standing desk (n=25) | 1 (4) | 1 (4) | 2 (8) | 1 (4) | 2 (8) | 4 (16) | 2 (8) | 8 (32) | 4 (16) | 7 (1.84) |
|  | Active workstation with underdesk stepper or pedaling device (n=27) | 1 (4) | 6 (22) | 2 (7) | 3 (11) | 7 (26) | 4 (15) | 2 (7) | 1 (4) | 1 (4) | 5 (1.63) |
|  | Active workstation equipped with treadmill (n=27) | 6 (22) | 9 (33) | 4 (15) | 2 (7) | 3 (11) | 1 (4) | 0 (0) | 1 (4) | 1 (4) | 2 (1.48) |
| **Guidelines, regulations, and restrictions** | | | | | | | | | | | |
|  | Self-monitoring sedentary and activity behaviors (ie, activity tracker or a diary log; n=27) | 0 (0) | 0 (0) | 0 (0) | 4 (15) | 2 (7) | 1 (4) | 5 (19) | 9 (33) | 6 (22) | 8 (1.30) |
|  | Create an action plan—increase standing breaks or replace sitting time, indicating the duration, frequency, and timing (eg, when the phone rings; n=27) | 0 (0) | 0 (0) | 1 (4) | 0 (0) | 1 (4) | 5 (19) | 8 (30) | 5 (19) | 7 (26) | 7 (1.11) |
|  | Standing while reading, answering phone calls, or conducting videoconferences (n=27) | 0 (0) | 0 (0) | 0 (0) | 1 (4) | 2 (7) | 4 (15) | 4 (15) | 5 (19) | 11 (41) | 8 (1.22) |
|  | Short breaks (5-10 min) approximately every 60 min of sitting time (n=27) | 1 (4) | 0 (0) | 0 (0) | 0 (0) | 6 (22) | 2 (7) | 6 (22) | 4 (15) | 8 (30) | 7 (1.48) |
|  | Setting tailored goals for reducing sitting time (n=27) | 0 (0) | 0 (0) | 1 (4) | 0 (0) | 1 (4) | 7 (26) | 8 (30) | 6 (22) | 4 (15) | 7 (1) |
|  | Scheduling (blocking) 5-10–min breaks between meetings on the calendar (n=27) | 0 (0) | 0 (0) | 2 (7) | 0 (0) | 3 (11) | 5 (19) | 7 (26) | 6 (22) | 4 (15) | 7 (1.22) |
|  | Incidental moving while reading, answering phone calls, or conducting videoconferences (n=27) | 1 (4) | 0 (0) | 0 (0) | 0 (0) | 2 (7) | 8 (30) | 5 (19) | 3 (11) | 8 (30) | 7 (1.37) |
|  | Active breaks, such as stretching, walking, or performing strengthening exercises for ≥10 min (n=27) | 0 (0) | 0 (0) | 1 (4) | 2 (7) | 4 (15) | 6 (22) | 6 (22) | 3 (11) | 5 (19) | 7 (1.37) |
|  | Team or individual activity challenges (n=27) | 0 (0) | 1 (4) | 1 (4) | 2 (7) | 4 (15) | 7 (26) | 5 (19) | 5 (19) | 2 (7) | 6 (1.33) |
|  | Scheduling (blocking) snack breaks on the calendar (n=26) | 0 (0) | 0 (0) | 3 (12) | 4 (15) | 2 (8) | 5 (19) | 4 (15) | 4 (15) | 4 (15) | 6 (1.65) |
|  | Active lunchtime, such as Pilates, yoga, walking, or cycling (n=27) | 0 (0) | 2 (7) | 2 (7) | 2 (7) | 4 (15) | 5 (19) | 4 (15) | 3 (11) | 5 (19) | 6 (1.74) |
|  | Short breaks (5-10 min) approximately every 40 min of sitting time (n=26) | 0 (0) | 1 (4) | 4 (15) | 1 (4) | 4 (15) | 5 (19) | 5 (19) | 3 (12) | 3 (12) | 6 (1.61) |
|  | Wellness coaches supporting the employees during breaks (n=27) | 0 (0) | 2 (7) | 2 (7) | 4 (15) | 5 (19) | 5 (19) | 5 (19) | 2 (7) | 2 (7) | 6 (1.55) |
|  | Short breaks (5-10 min) approximately every 30 min of sitting time (n=27) | 1 (4) | 4 (15) | 2 (7) | 5 (19) | 5 (19) | 3 (11) | 4 (15) | 1 (4) | 2 (7) | 5 (1.74) |
|  | Blocking the screen or keyboard for breaking up sitting time unless conducting a meeting (n=26) | 6 (23) | 2 (8) | 3 (12) | 1 (4) | 4 (15) | 4 (15) | 3 (12) | 2 (8) | 1 (4) | 5 (2.19) |
| **Communication and social support** | | | | | | | | | | | |
|  | Feedback on activity progress and goal achievement (n=27) | 0 (0) | 0 (0) | 0 (0) | 1 (4) | 1 (4) | 3 (11) | 11 (41) | 10 (37) | 1 (4) | 7 (1.74) |
|  | Providing information to increase awareness and knowledge of the dangers associated with prolonged sedentary behavior and the potential benefits of reducing it or breaking it up (n=27) | 0 (0) | 0 (0) | 1 (4) | 1 (4) | 2 (7) | 3 (11) | 6 (22) | 4 (15) | 10 (37) | 8 (1.37) |
|  | Awards, rewards, or incentives to achieve goals or recommendations (n=27) | 0 (0) | 0 (0) | 0 (0) | 2 (7) | 1 (4) | 5 (19) | 10 (37) | 4 (15) | 5 (19) | 7 (1) |
|  | Information and support about the strategies and goals and reminders (n=27) | 0 (0) | 0 (0) | 0 (0) | 1 (4) | 1 (4) | 7 (26) | 8 (30) | 4 (15) | 6 (22) | 7 (1.04) |
|  | Real-time records or feedback (n=27) | 0 (0) | 0 (0) | 0 (0) | 1 (4) | 0 (0) | 9 (33) | 2 (7) | 12 (44) | 3 (11) | 8 (1) |
|  | Point-of-choice or point-of-decision prompts (n=26) | 0 (0) | 1 (4) | 0 (0) | 1 (4) | 3 (12) | 4 (15) | 11 (42) | 4 (15) | 2 (8) | 7 (1) |
|  | Self-selected reminders to achieve goals (n=27) | 0 (0) | 0 (0) | 0 (0) | 0 (0) | 2 (7) | 9 (33) | 4 (15) | 9 (33) | 3 (11) | 7 (1.04) |
|  | Motivational messages from managers (n=27) | 0 (0) | 2 (7) | 0 (0) | 1 (4) | 2 (7) | 6 (22) | 6 (22) | 7 (26) | 3 (11) | 7 (1.33) |
|  | Activity demonstrations to perform during the breaks (n=27) | 0 (0) | 0 (0) | 1 (4) | 2 (7) | 2 (7) | 7 (26) | 8 (30) | 4 (15) | 3 (11) | 7 (1.15) |
|  | Social networking for sharing experiences (n=27) | 0 (0) | 2 (7) | 0 (0) | 2 (7) | 3 (11) | 6 (22) | 7 (26) | 5 (19) | 2 (7) | 7 (1.37) |
|  | Social comparison (n=25) | 0 (0) | 0 (0) | 3 (12) | 5 (20) | 4 (16) | 2 (8) | 7 (28) | 4 (16) | 0 (0) | 6 (1.52) |
|  | Competition among peers (n=27) | 0 (0) | 1 (4) | 3 (11) | 4 (15) | 7 (26) | 2 (7) | 6 (22) | 4 (15) | 0 (0) | 5 (1.44) |

^a^MAD-M: mean absolute deviation from the median.
