# Supplementary material for: Development of Digital Strategies for Reducing Sedentary Behavior in a Hybrid Office Environment: Modified Delphi Study
Source: JMIR Hum Factors. 2025 Apr 8;12:e59405. doi: 10.2196/59405 (PMC12015347; doi:10.2196/59405)
Supplement: Multimedia Appendix 4 [file humanfactors_v12i1e59405_app4.docx]

**Multimedia Appendix 4.** Usefulness of behavioral intervention technology (BIT) elements (results of round 1).

| BIT element to communicate information | | | | Level of usefulness, n (%) | | | | | | | | | Median (MAD-M^a^) |
| --- | --- | --- | --- | --- | --- | --- | --- | --- | --- | --- | --- | --- | --- |
|  | | | | Not useful | | | Neutrally useful | | | Highly useful | | |  |
|  | | | | Score of 1 | Score of 2 | Score of 3 | Score of 4 | Score of 5 | Score of 6 | Score of 7 | Score of 8 | Score of 9 |  |
|  | | | | | | | | | | | | | |
| **Messaging and social support** | | | | | | | | | | | | | |
|  | | Social challenge features (cooperative activities, eg, reach 10,000 steps among all members of the department; n=27) | | 0 (0) | 0 (0) | 0 (0) | 1 (4) | 1 (4) | 5 (19) | 8 (30) | 8 (30) | 4 (15) | 7 (1.22) |
|  | | Gamification features (competition among colleagues; n=26) | | 0 (0) | 1 (4) | 1 (4) | 2 (8) | 3 (12) | 5 (19) | 8 (31) | 4 (15) | 2 (8) | 7 (1.78) |
|  | | Chats (eg, WhatsApp groups; n=27) | | 0 (0) | 0 (0) | 3 (11) | 6 (22) | 4 (15) | 1 (4) | 5 (19) | 6 (22) | 2 (7) | 6 (2) |
|  | | Emails (n=27) | | 0 (0) | 2 (7) | 5 (19) | 2 (7) | 3 (11) | 6 (22) | 6 (22) | 1 (4) | 2 (7) | 6 (1.78) |
|  | | Calls (n=27) | | 4 (15) | 1 (4) | 2 (7) | 3 (11) | 7 (26) | 4 (15) | 3 (11) | 2 (7) | 1 (4) | 5 (1.85) |
|  | | Forums (n=26) | | 0 (0) | 4 (15) | 3 (12) | 4 (15) | 4 (15) | 5 (19) | 4 (15) | 0 (0) | 2 (8) | 5 (1.77) |
| **Push notifications** | | | | | | | | | | | | |  |
|  | | App interface—text and sound and vibration in smartphones (n=27) | | 0 (0) | 2 (7) | 0 (0) | 3 (11) | 2 (7) | 5 (19) | 7 (26) | 6 (22) | 2 (7) | 7 (1.41) |
|  | | Application interface—computer screen notification in desktop computers (n=27) | | 0 (0) | 1 (4) | 0 (0) | 2 (7) | 4 (15) | 6 (22) | 8 (30) | 5 (19) | 1 (4) | 7 (1.18) |
|  | | SMS text messages (n=27) | | 1 (4) | 3 (11) | 2 (7) | 1 (4) | 3 (11) | 5 (19) | 8 (30) | 2 (7) | 2 (7) | 6 (1.63) |
|  | | Chats (n=26) | | 2 (8) | 0 (0) | 3 (12) | 4 (15) | 2 (8) | 5 (19) | 6 (23) | 2 (8) | 2 (8) | 6 (1.73) |
|  | | Emails (n=27) | | 0 (0) | 4 (15) | 3 (11) | 5 (19) | 2 (7) | 7 (26) | 4 (15) | 1 (4) | 1 (4) | 5 (1.63) |
| **Information delivery** | | | | | | | | | | | | | |
|  | | Combination of media^b^ (n=24) | | 1 (4) | 0 (0) | 3 (12) | 1 (4) | 2 (8) | 3 (12) | 4 (17) | 7 (29) | 3 (12) | 7 (1.71) |
|  | | Videos (n=27) | | 1 (4) | 0 (0) | 1 (4) | 4 (15) | 4 (15) | 4 (15) | 10 (37) | 2 (7) | 1 (4) | 6 (1.33) |
|  | | App interface—smartphones (n=27) | | 0 (0) | 3 (11) | 0 (0) | 6 (22) | 4 (15) | 2 (7) | 5 (19) | 6 (22) | 1 (4) | 6 (1.78) |
|  | | Images (n=27) | | 0 (0) | 1 (4) | 2 (7) | 6 (22) | 1 (4) | 6 (22) | 6 (22) | 4 (15) | 1 (4) | 6 (1.44) |
|  | | Websites (n=27) | | 1 (4) | 1 (4) | 6 (22) | 2 (7) | 1 (4) | 5 (19) | 4 (15) | 7 (26) | 0 (0) | 6 (1.81) |
|  | | Application interface—desktops (n=27) | | 0 (0) | 3 (11) | 0 (0) | 5 (19) | 5 (19) | 5 (19) | 4 (15) | 4 (15) | 1 (4) | 6 (1.55) |
|  | | Emails (n=26) | | 0 (0) | 4 (15) | 5 (19) | 5 (19) | 3 (12) | 2 (8) | 6 (23) | 1 (4) | 0 (0) | 4 (1.61) |
|  | | SMS text messages (n=27) | | 1 (4) | 5 (19) | 0 (0) | 2 (7) | 8 (30) | 4 (15) | 5 (19) | 1 (4) | 1 (4) | 5 (1.55) |
|  | | Audios (n=27) | | 1 (4) | 1 (4) | 5 (19) | 3 (11) | 5 (19) | 6 (22) | 4 (15) | 2 (7) | 0 (0) | 5 (1.41) |
| **Report and visualization** | | | | | | | | | | | | | |
|  | | Real-time data via app interface (n=27) | | 0 (0) | 1 (4) | 2 (7) | 2 (7) | 0 (0) | 3 (11) | 7 (26) | 6 (22) | 6 (22) | 7 (1.48) |
|  | | Data summary via app interface (n=27) | | 0 (0) | 1 (4) | 1 (4) | 1 (4) | 3 (11) | 7 (26) | 2 (7) | 4 (15) | 8 (30) | 7 (1.67) |
|  | | Data summary via email or SMS text message (n=27) | | 0 (0) | 2 (7) | 3 (11) | 4 (15) | 2 (7) | 8 (30) | 3 (11) | 4 (15) | 1 (4) | 6 (1.52) |
|  | | Data summary via website (n=27) | | 0 (0) | 2 (7) | 4 (15) | 3 (11) | 5 (19) | 7 (26) | 5 (19) | 1 (4) | 0 (0) | 5 (1.37) |
| **BIT elements to track activity** | | | | | | | | | | | | | |
|  | | **Digital log (manual entry)** | | | | | | | | | | | |
|  | |  | Mobile phone diary (n=27) | 0 (0) | 1 (4) | 2 (7) | 1 (4) | 2 (7) | 4 (15) | 6 (22) | 8 (30) | 3 (11) | 7 (1.41) |
|  | |  | Computer software diary (n=27) | 0 (0) | 1 (4) | 5 (19) | 2 (7) | 5 (19) | 5 (19) | 6 (22) | 2 (7) | 1 (4) | 6 (1.52) |
|  | |  | Web-based questionnaire (n=27) | 2 (7) | 2 (7) | 6 (22) | 2 (7) | 4 (15) | 5 (19) | 4 (15) | 2 (7) | 0 (0) | 5 (1.74) |
|  | | **Passive data collection** | | | | | | | | | | | |
|  | |  | Wrist-based activity tracker (n=26) | 0 (0) | 3 (12) | 1 (4) | 2 (8) | 1 (4) | 2 (8) | 7 (27) | 5 (19) | 5 (19) | 7 (1.69) |
|  | |  | Smartphone sensors (n=26) | 0 (0) | 2 (8) | 0 (0) | 4 (15) | 3 (12) | 4 (15) | 6 (23) | 4 (15) | 3 (12) | 7 (1.61) |
|  | |  | Leg-based activity tracker (n=26) | 2 (8) | 1 (4) | 3 (12) | 0 (0) | 1 (4) | 8 (31) | 4 (15) | 6 (23) | 1 (4) | 6 (1.65) |
|  | |  | Waist-based activity tracker (n=26) | 1 (4) | 5 (19) | 4 (15) | 1 (4) | 4 (15) | 4 (15) | 5 (19) | 2 (8) | 0 (0) | 5 (1.85) |
|  | |  | Cushions on chairs (n=24) | 0 (0) | 2 (8) | 5 (21) | 3 (12) | 2 (8) | 5 (21) | 2 (8) | 3 (12) | 2 (8) | 6 (1.87) |
|  | |  | Electronic workstation (n=25) | 0 (0) | 3 (12) | 3 (12) | 1 (4) | 2 (8) | 9 (36) | 2 (8) | 2 (8) | 3 (12) | 6 (1.6) |
|  | |  | Computer software (n=26) | 0 (0) | 2 (8) | 6 (23) | 2 (8) | 4 (15) | 6 (23) | 3 (12) | 2 (8) | 1 (4) | 5 (1.61) |

^a^MAD-M: mean absolute deviation from the median.

^b^New items: mixture of videos, online information, and images within a website; app and social media; videos and images; audio and video combination with an explanation (educational purposes); moving images on a website; smartphone and desktop with website or app for background information; personalized messages and apps; reminders combined with an app; reminders combined with social challenges; forum for general information and smartphone messages (eg, SMS text messages) for specific tailored information; forum for general information and app for specific tailored information; forum for general information and email for specific tailored information; website for general information and smartphone messages (eg, SMS text messages) for specific tailored information; website for general information and email for specific tailored information; and website for general information and app for specific tailored information.
