# Supplementary material for: Development of Digital Strategies for Reducing Sedentary Behavior in a Hybrid Office Environment: Modified Delphi Study
Source: JMIR Hum Factors. 2025 Apr 8;12:e59405. doi: 10.2196/59405 (PMC12015347; doi:10.2196/59405)
Supplement: Multimedia Appendix 5 [file humanfactors_v12i1e59405_app5.docx]

**Multimedia Appendix 5.** Matching behavioral intervention technology (BIT) elements with work strategies on which consensus was achieved (round 3 results).

| Strategy and BIT element | | Respondents, n (%) |
| --- | --- | --- |
| **Feedback on activity progress and goal achievement** | | |
|  | Gamification features | 3 (15.8) |
|  | SMS text messages | 3 (15.8) |
|  | App interface—smartphone | 4 (21.1) |
|  | Images | 1 (5.26) |
|  | Combination of media | 1 (5.26) |
|  | Wrist-based activity tracker | 3 (15.8) |
|  | Real-time data | 1 (5.26) |
|  | Data summary | 3 (15.8) |
| **Create an action plan—increase standing breaks or replace sitting time, how long, how often, when, and how (eg, when the phone rings)** | | |
|  | Chat | 2 (10.5) |
|  | App interface—smartphone | 6 (31.6) |
|  | Application interface—desktop | 2 (10.5) |
|  | Videos | 1 (5.26) |
|  | Websites | 2 (10.5) |
|  | Combination of media | 2 (10.5) |
|  | Computer software | 3 (15.8) |
|  | —^a^ | 1 (5.26) |
| **Standing while reading, answering phone calls, or conducting videoconferences** | | |
|  | Gamification features | 2 (10.5) |
|  | SMS text messages | 1 (5.26) |
|  | App interface—smartphone | 6 (31.6) |
|  | Application interface—desktop | 3 (15.8) |
|  | Images | 1 (5.26) |
|  | Websites | 1 (5.26) |
|  | Leg-based activity tracker | 2 (10.5) |
|  | — | 3 (15.8) |
| **Providing information to increase awareness and knowledge of the dangers associated with prolonged sedentary behavior and the potential benefits of reducing it or breaking it up** | | |
|  | Gamification features | 2 (10.5) |
|  | App interface—smartphone | 3 (15.8) |
|  | Application interface—desktop | 2 (10.5) |
|  | Videos | 3 (15.8) |
|  | Websites | 4 (21.1) |
|  | Combination of media | 5 (26.3) |
| **Self-monitoring sedentary and activity behaviors (ie, activity tracker or a diary log)** | | |
|  | App interface—smartphone | 1 (5.26) |
|  | Mobile phone diary | 1 (5.26) |
|  | Leg-based activity tracker | 3 (15.8) |
|  | Wrist-based activity tracker | 12 (63.2) |
|  | Smartphone sensors | 1 (5.26) |
|  | — | 1 (5.26) |
| **Short breaks (5-10 min) approximately every 60 min of sitting time** | | |
|  | SMS text messages | 2 (10.5) |
|  | App interface—smartphone | 3 (15.8) |
|  | Application interface—desktop | 6 (31.6) |
|  | Wrist-based activity tracker | 2 (10.5) |
|  | Computer software | 3 (15.8) |
|  | Real-time data | 2 (10.5) |
|  | — | 1 (5.26) |
| **Scheduling (blocking) 5-10–min breaks between meetings on the calendar** | | |
|  | Gamification features | 1 (5.26) |
|  | Chat | 1 (5.26) |
|  | App interface—smartphone | 2 (10.5) |
|  | Application interface—desktop | 3 (15.8) |
|  | Combination of media | 1 (5.26) |
|  | Mobile phone diary | 1 (5.26) |
|  | Computer software | 9 (47.4) |
|  | Real-time data | 1 (5.26) |
| **Information and support about the strategies and goals and reminders** | | |
|  | Gamification features | 1 (5.26) |
|  | Chat | 1 (5.26) |
|  | SMS text messages | 1 (5.26) |
|  | App interface—smartphone | 5 (26.3) |
|  | Application interface—desktop | 4 (21.1) |
|  | Combination of media | 5 (26.3) |
|  | Computer software | 1 (5.26) |
|  | Real-time data | 1 (5.26) |
| **Setting tailored goals for reducing sitting time** | | |
|  | Gamification features | 1 (5.26) |
|  | App interface—smartphone | 8 (42.1) |
|  | Application interface—desktop | 3 (15.8) |
|  | Websites | 1 (5.26) |
|  | Combination of media | 1 (5.26) |
|  | Mobile phone diary | 1 (5.26) |
|  | Wrist-based activity tracker | 1 (5.26) |
|  | Computer software | 1 (5.26) |
|  | Real-time data | 1 (5.26) |
|  | — | 1 (5.26) |
| **Activity demonstrations to perform during the breaks** | | |
|  | Gamification features | 1 (5.26) |
|  | App interface—smartphone | 4 (21.1) |
|  | Application interface—desktop | 1 (5.26) |
|  | Videos | 9 (47.4) |
|  | Images | 1 (5.26) |
|  | Combination of media | 2 (10.5) |
|  | — | 1 (5.26) |
| **Height-adjustable desks or desk platforms** | | |
|  | Chat | 1 (5.26) |
|  | SMS text messages | 1 (5.26) |
|  | App interface—smartphone | 1 (5.26) |
|  | Videos | 1 (5.26) |
|  | Combination of media | 2 (10.5) |
|  | Leg-based activity tracker | 1 (5.26) |
|  | Computer software | 1 (5.26) |
|  | — | 11 (57.9) |
| **Relocation of home office supplies (eg, bins and printers)** | | |
|  | Chat | 1 (5.26) |
|  | SMS text messages | 1 (5.26) |
|  | App interface—smartphone | 2 (10.5) |
|  | Application interface—desktop | 1 (5.26) |
|  | Videos | 1 (5.26) |
|  | Images | 1 (5.26) |
|  | Websites | 2 (10.5) |
|  | Combination of media | 4 (21.1) |
|  | Real-time data | 1 (5.26) |
|  | — | 5 (26.3) |
| **Incidental moving while reading, answering phone calls, or conducting videoconferences** | | |
|  | Gamification features | 2 (10.5) |
|  | App interface—smartphone | 2 (10.5) |
|  | Application interface—desktop | 2 (10.5) |
|  | Videos | 2 (10.5) |
|  | Images | 1 (5.26) |
|  | Combination of media | 3 (15.8) |
|  | Wrist-based activity tracker | 2 (10.5) |
|  | Smartphone sensors | 2 (10.5) |
|  | Real-time data | 1 (5.26) |
|  | — | 2 (10.5) |
| **Awards, rewards, or incentives to achieve goals or recommendations** | | |
|  | Gamification features | 8 (42.1) |
|  | Chat | 1 (5.26) |
|  | App interface—smartphone | 4 (21.1) |
|  | Application interface—desktop | 1 (5.26) |
|  | Combination of media | 2 (10.5) |
|  | Data summary | 1 (5.26) |
|  | — | 2 (10.5) |
| **Standing desk** | | |
|  | Gamification features | 1 (5.26) |
|  | Chat | 1 (5.26) |
|  | App interface—smartphone | 1 (5.26) |
|  | Videos | 1 (5.26) |
|  | Images | 1 (5.26) |
|  | Combination of media | 2 (10.5) |
|  | Leg-based activity tracker | 1 (5.26) |
|  | Computer software | 1 (5.26) |
|  | Real-time data | 1 (5.26) |
|  | — | 9 (47.4) |
| **Point-of-choice or point-of-decision prompts** | | |
|  | Chat | 1 (5.26) |
|  | SMS text messages | 1 (5.26) |
|  | App interface—smartphone | 3 (15.8) |
|  | Images | 2 (10.5) |
|  | Combination of media | 2 (10.5) |
|  | Wrist-based activity tracker | 1 (5.26) |
|  | Smartphone sensors | 1 (5.26) |
|  | Computer software | 3 (15.8) |
|  | Real-time data | 1 (5.26) |
|  | — | 4 (21.1) |
| **Workstation accessories (seated footrests, standing footrests, or sit-stand antifatigue mats)** | | |
|  | Chat | 2 (10.5) |
|  | App interface—desktop | 1 (5.26) |
|  | Videos | 2 (10.5) |
|  | Images | 1 (5.26) |
|  | Combination of media | 3 (15.8) |
|  | Wrist-based activity tracker | 1 (5.26) |
|  | — | 9 (47.4) |
| **Motivational messages from managers** | | |
|  | Chat | 2 (10.5) |
|  | SMS text messages | 1 (5.26) |
|  | App interface—smartphone | 4 (21.1) |
|  | Application interface—desktop | 1 (5.26) |
|  | Videos | 3 (15.8) |
|  | Combination of media | 2 (10.5) |
|  | Mobile phone diary | 1 (5.26) |
|  | Data summary | 1 (5.26) |
|  | — | 4 (21.1) |

^a^Not applicable.
